# SEX STEROID HORMONES AND AGE AT DEATH—A MENDELIAN APPROACH

**DOI:** 10.1093/geroni/igad104.2494

**Published:** 2023-12-21

**Authors:** Cynthia Kusters, Kimberly Paul, Beate Ritz

**Affiliations:** University of California, Los Angeles, Los Angeles, California, United States; University of California, Los Angeles, Los Angeles, California, United States; University of California, Los Angeles, Los Angeles, California, United States

## Abstract

Lower concentrations of testosterone have been associated with increased mortality and shorter lifespan in observational studies. An association between androgens and decreased epigenetic age was identified among men. Here, we assessed the association between sex steroid hormones and parental age at death using a Mendelian randomization (MR) approach. An inverse-variance weighting (IVW) MR analysis was performed using effect estimates from external GWAS summary statistics. We included independent variants (linkage disequilibrium R2< 0.001) and a P-value threshold of 5x10-8. Sensitivity analysis included changing the P-value thresholds, MR method and multivariable MR analysis. An increase in androsterone sulfate and DHEAS were associated with a lower age at death for both fathers and mothers (-0.04/-0.05 years, P-value: 7e-4/7e-4), as well as the combined parental age at death (-0.06 years, P-value:5e-4). In addition, an increase in SHBG was associated with a higher age at death (0.06 years, P-value:7e-5). We did not find an association with estradiol or testosterone and age at death. Sensitivity analysis showed consistent findings. Our study could suggest that a higher concentration of androgens, specifically androsterone sulfate and DHEAS, decrease lifespan. This is contradictory to observational studies and deserves further attention. However, the SNPs associated with these androgens are also strongly associated with body mass index (BMI). Adjusting for BMI in the MR models removes the association between sex hormones and age at death. We are currently performing more analyses to shed light on this association.

